# Jejunal Atresia With a Rare Association: A Case Report and Literature Review

**DOI:** 10.7759/cureus.58141

**Published:** 2024-04-12

**Authors:** Osama Qumsieh, Lina Qawasmeh, Reem Jaradat, Amani Rashideh, Danya Juba, Basel A Zaben

**Affiliations:** 1 Pediatric Surgery, Palestine Polytechnic University, Hebron, PSE; 2 Faculty of Medicine and Health Sciences, Palestine Polytechnic University, Hebron, PSE; 3 Medicine, Palestine Polytechnic University, Hebron, PSE; 4 Surgery, Al-Quds University, Ramallah, PSE

**Keywords:** case report, intestinal obstruction, double bubble sign, colostomy, jejunoileal atresia, rectal stenosis

## Abstract

Jejunoileal atresia, a common cause of neonatal intestinal obstruction, typically manifests shortly after birth. This case report highlights a rare instance of a late preterm female neonate presenting with type 4 jejunoileal atresia along with proximal rectal atresia, an exceedingly uncommon combination. Initial symptoms included bilious emesis and failure to pass meconium, leading to surgical correction of jejunoileal atresia. However, postoperative complications, including vomiting and jaundice, prompted further investigation, revealing rectal atresia during a fluoroscopic study on day 29. Subsequent surgery was required to address the rectal atresia, resulting in additional challenges such as short bowel syndrome and infection. The complexity of diagnosis and management underscores the importance of thorough evaluation of the lower gastrointestinal tract in neonates with jejunoileal atresia to prevent misdiagnosis and reduce the need for multiple surgeries. Rectal atresia, which is a very rare anorectal abnormality, in combination with jejunoileal atresia is considered an incredibly unusual, exceptionally unique case; as to our knowledge, no similar presentation had previously occurred. Prompt identification and simultaneous treatment of both conditions can help mitigate complications, minimize the risk of necrosis and perforation, and improve overall outcomes. Comprehensive management strategies that encompass thorough diagnostic evaluation and coordinated surgical interventions are crucial for optimizing the care of neonates with complex intestinal malformations, ensuring timely resolution of symptoms, and reducing long-term morbidity.

## Introduction

Jejunoileal atresia is a generally widespread cause of neonatal intestinal obstruction, which happens secondary to intrauterine mesenteric vascular accidents, estimated to occur in every 2.1 per 10,000 births [1]. The vast majority of these patients present within the first 24 hours of life, and the path henceforth is fairly straightforward. Both cystic fibrosis and heart abnormality cases are widely associated with jejunoileal atresia (JIA) [2]. However, rectal atresia, which is considered a very rare anorectal malformation, in combination with jejunoileal atresia is exceedingly rare and exceptionally unique, and to our knowledge, no similar instances have been reported in the medical literature before [3]. Both defects arise from unrelated embryological development and have their own set of clinical features [4]. A causal relationship has yet to be elicited and clarified. The combination thus provides not just an etiological mystery but also an exceptional nature of this diagnostic challenge and highlights the novelty of the therapeutic approach required. As jejunoileal atresia can affect multiple lengths of the bowel, the severity increases with advancing type of atresia, which can be classified into four types: Type I atresia is defined as the presence of luminal webs with mural continuity. Type II atresia is a fibrous cord that connects two blind ends. Type IIIa atresia has two unrelated ends with a mesenteric gap. Type IIIb, also known as "apple peel atresia", is distinguished by two separable ends, with a substantial mesenteric defect. Features of Type IV consist of multiple atretic segments (resembling a string of sausages) [5,6].

The pathogenesis of such identified rare type proximal rectal atresia, which is defined as atresia with a central holed septal defect, is thought to be due to either thrombosis of vessels in the presence of intrauterine infection causing atresia of the already formed rectum or vascular accidents that happen between 13 and 14 weeks of gestation [7]. The main usual clinical picture that patients presented with is abdominal distension and failure to pass meconium beyond 48 hours, as a similar picture presented in our case [8]. Reaching the exact diagnosis of such a unique case is achieved by several multiple radiological assessments, with proper surgical repair being the treatment of choice. Here, in our case, we report an extraordinary unprecedented clinical scenario of Type 4 jejunoileal atresia and proximal rectal stenosis in combination, presented in a late preterm neonate. This case has been reported in line with Surgical Case Report (SCARE) criteria [9].

## Case presentation

A four-hour-old premature female neonate patient presented to our hospital due to prenatal evidence of intestinal obstruction with complaints of repeated episodes of bilious emesis and absent passage of meconium since birth. She was delivered at the government hospital at 36 +3 weeks of gestational age by cesarean section (due to previous C/S) for a gravida 8, para 6, abortion 2 (G8 P6 A2) mother. She weighed 2,600 g at birth with a length of 45 cm and a head circumference of 33 cm. At the 24th week of pregnancy, during routine pregnancy checks, severe polyhydramnios was evident, and a detailed ultrasound was done and revealed evidence of intestinal obstruction. The baby was referred to our hospital on the same day of delivery for surgical evaluation. At the time of admission, the neonate's vital signs were as follows: a temperature of 35.5 degrees Celsius, in the re-evaluation it became 36.3 degrees Celsius which confirmed the idea that the initial temperature result was caused by a drop in the temperature of the surrounding environment during the child’s transport by ambulance to the hospital, a respiratory rate of 57 breaths per minute, and a heart rate of 133 beats per minute. The mean arterial pressure (MAP) was 44 mmHg, and the oxygen saturation (SPO_2_) was 100% without supplemental oxygen. The glucose level was measured at 76 mg/dl. Additionally, the patient underwent various tests. The venous blood gas (VBG) analysis revealed a pH of 7.27, partial pressure of carbon dioxide (pCO_2_) of 34 mmHg, partial pressure of oxygen (pO_2_) of 57 mmHg, and a bicarbonate (HCO_3_) level of 16.6 mEq/L. The total serum bilirubin (TSB) level was 6.5 mg/dl; these tests are shown in Table 1. Abdominal examination revealed a mildly distended, soft, and non-tender abdomen. Bowel sounds were absent. On perineal examination, the anal opening was normal-looking, and the anal canal and lower rectum are surrounded by a normally developed sphincter. Chest auscultation reported bilateral good air entry with no added sound. Mouth examination revealed a small, whitish, round-shaped lesion on the hard and soft palate that was diagnosed as a benign lesion and did not require any evaluation or treatment. Routine blood work and radiography were done. The complete blood parameters were within the normal range. Renal function tests were normal, and an X-ray of the erect abdomen demonstrated a double bubble sign.

**Table 1:** Laboratory tests done on admission HCO_3_: bicarbonate.

Lab Test	Normal Range	Result
Glucose	70-150 mg/dl	76 mg/dl
Venous pH	7.35-7.45	7.27
Venous CO_2_	35-45 mmHg	34 mmHg
Venous O_2_	45-65 mmHg	57 mmHg
Venous HCO_3_	22-26 mEq/L	16.6 mEq/ L
Total serum bilirubin (TSB)	Less than 1.2 mg/dl	6.5 mg/dl

The intestinal obstruction is known to be linked to a number of congenital anomalies, the most significant of which are cardiac and neurological disorders. An echo test was conducted with a normal result, and the results of the neurological exam showed that the baby was active, had good power and tone, and had no aberrant movements or apnea.

The preoperative protocol, which involved maintaining adequate hydration, restoring lab values to normal, and decompressing the intestines with the insertion of a nasogastric tube, was done. Following assessment, the baby was sent for an exploratory laparotomy on the second day of life with the goal of surgically repairing the intestinal blockage. A large horizontal scar on the right side of the abdomen from the laparotomy incision was made. The intestines were examined to determine the site of any obstruction due to congenital atresia. Type 4 jejunal atresia was discovered, which involved a large segment of the small bowel, starting from the Treitz ligament to the beginning of the ileum. The patient underwent duodeno-ileal anastomosis. The perfusion of the gut was normal on exposure, with no perforation, serosal tear, or any other anomaly such as volvulus associated with malrotation found.

**Figure 1:**
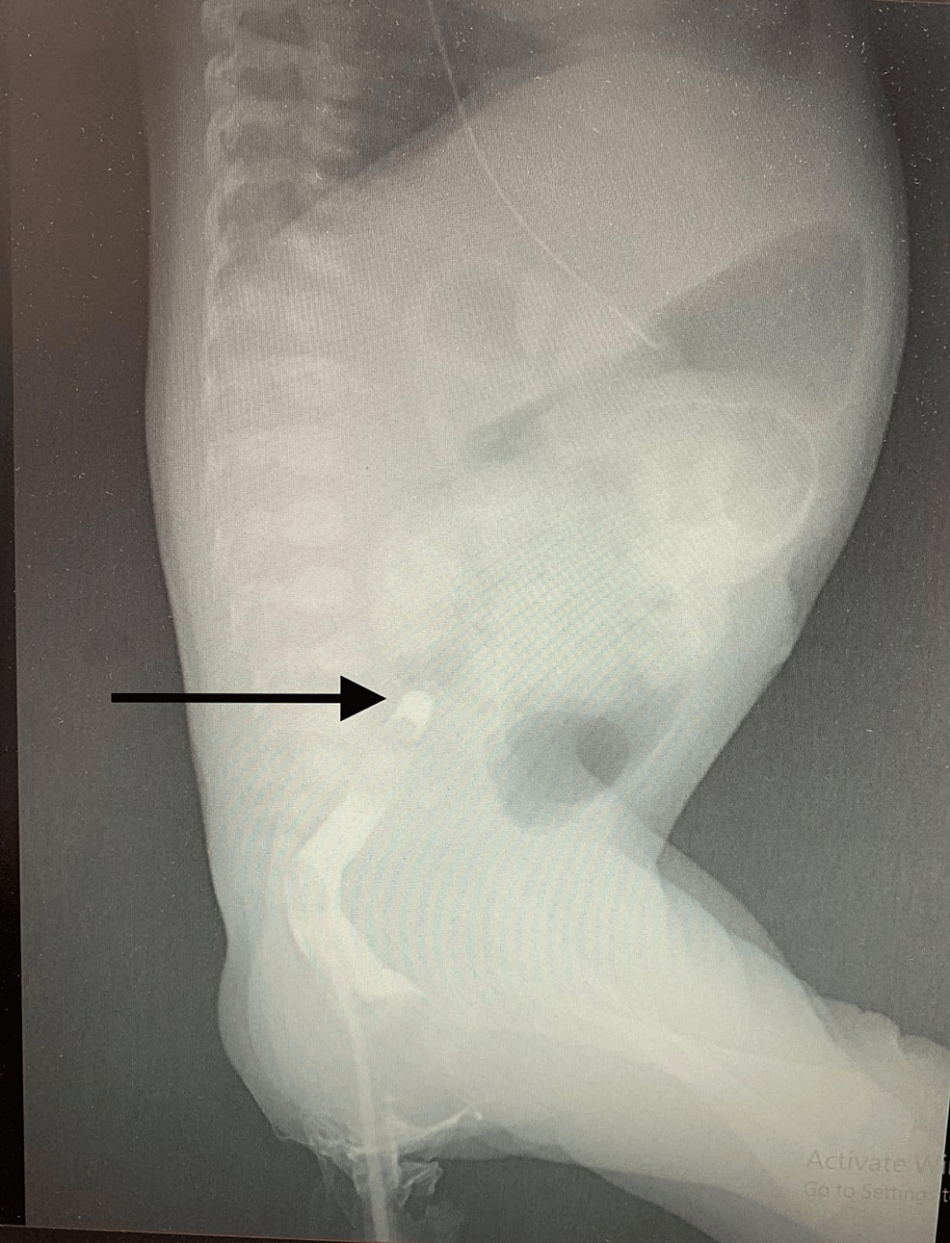
Fluorography lateral view of the abdomen Black arrow points to the site of stoppage of contrast at the site of rectal stenosis.

Bilious emesis as a clinical presentation is compatible with the finding of proximal bowel obstruction. So the operation was ended, and the patient’s abdomen was closed. After that, the patient was sent to the newborn critical care unit (NICU) for additional monitoring and care. The child's vital signs were stable, and all postoperative laboratory measurements and parameters were within acceptable ranges. IV analgesics, hydration, and antibiotics were given. Two days after the surgery, the NICU nurses recorded the presence of a small amount of stool, which had occurred two to three times. Therefore, oral feeding by orogastric tube was started. The patient then began to suffer from whitish-colored vomiting in large quantities. Oral feeding was stopped, and she returned to complete parental nutrition. On the eighth day of her life, she began to exhibit jaundice (TSB = 18.6, level 19), as shown in Table 2. Phototherapy was administered, and the condition improved over time. Routine blood work and radiography were done. The complete blood parameters were within the normal range. Renal function tests were normal, and an X-ray of the erect abdomen was unremarkable. In addition to normal bowel sounds on physical examination, a cystic fibrosis screening test was conducted using a venous blood sample to consider other potential causes for the failure to pass meconium, particularly those related to intestinal atresia as seen in our case. The test utilized the immunoreactive trypsinogen (IRT) method, and the results came back negative. However, the specific value of the test was not recorded in the patient's report. These findings encourage that the patient may improve with time. However, with the continued occurrence of abdominal bloating and vomiting of any milk taken and the absence of the normal amount of meconium in the pad, with no improvement in patient state, a fluoroscopic study of the lower gastrointestinal (GI) was performed on 29th day of the patient life to show that there is an obstruction a few centimeter proximal to the dentate line, as shown in Figure 1, which gave a suggestion for the existence of a type of congenital rectal atresia.

**Table 2:** Postoperative laboratory tests

Lab Test	Normal Range	Result
Total serum bilirubin (TSB)	Up to 15 mg/dl	18.6 mg/dl

**Figure 2:**
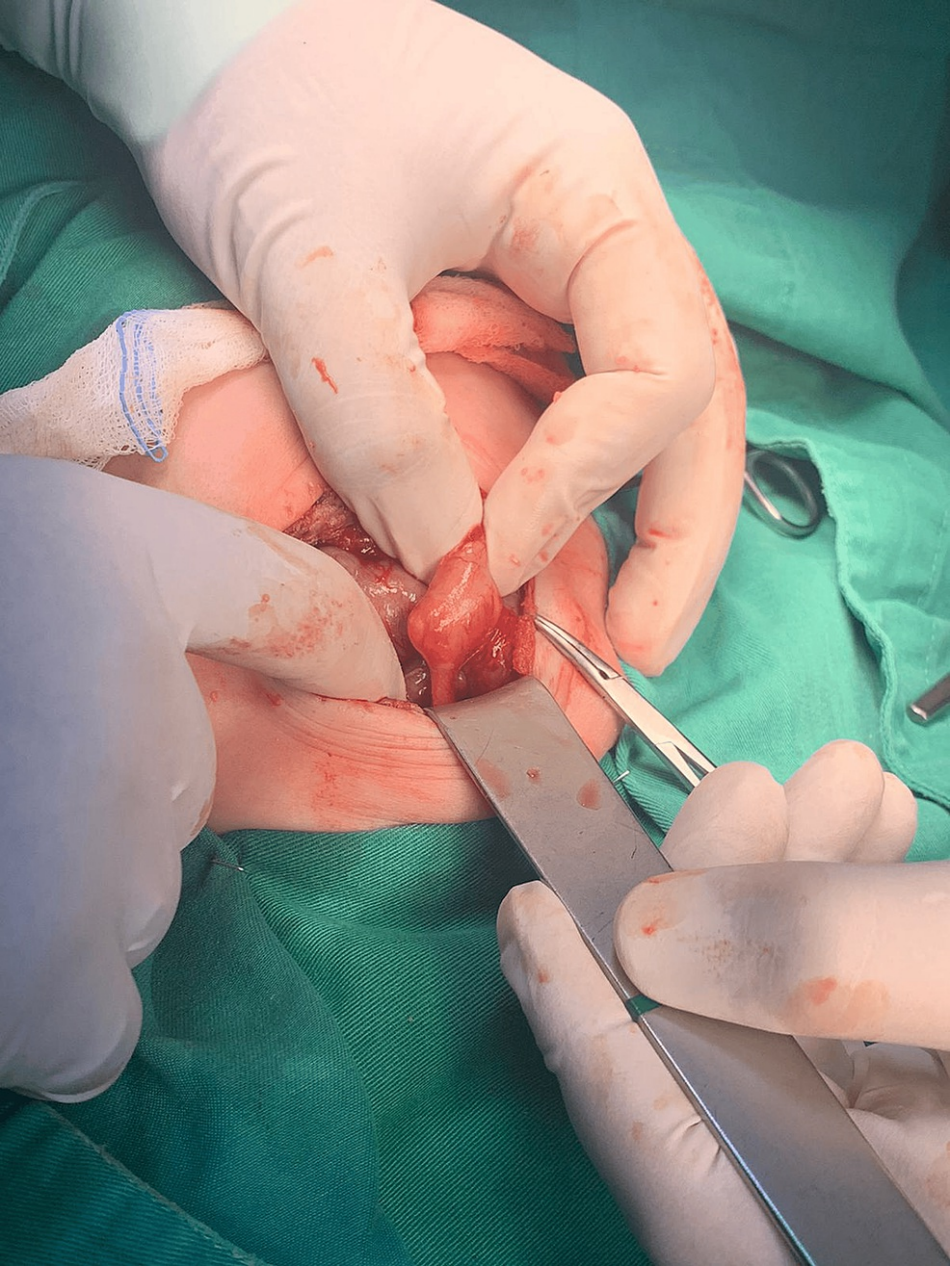
Intraoperative photograph showing rectal stenosis

**Figure 3:**
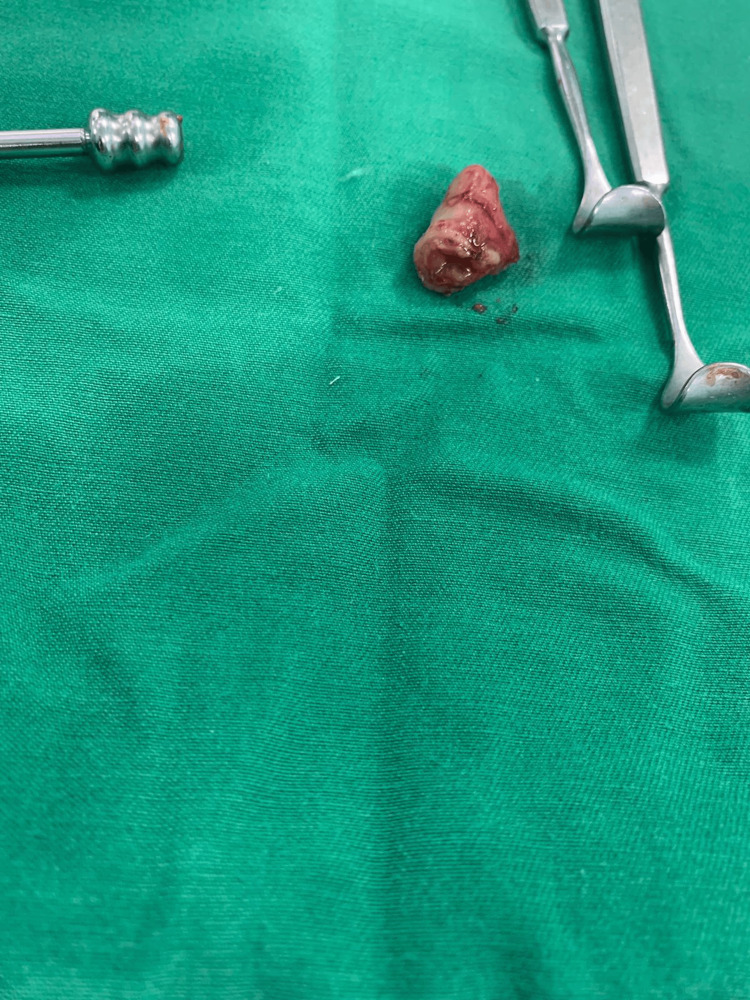
Intraoperative photograph showing the excision of the atretic segment of rectum

**Figure 4:**
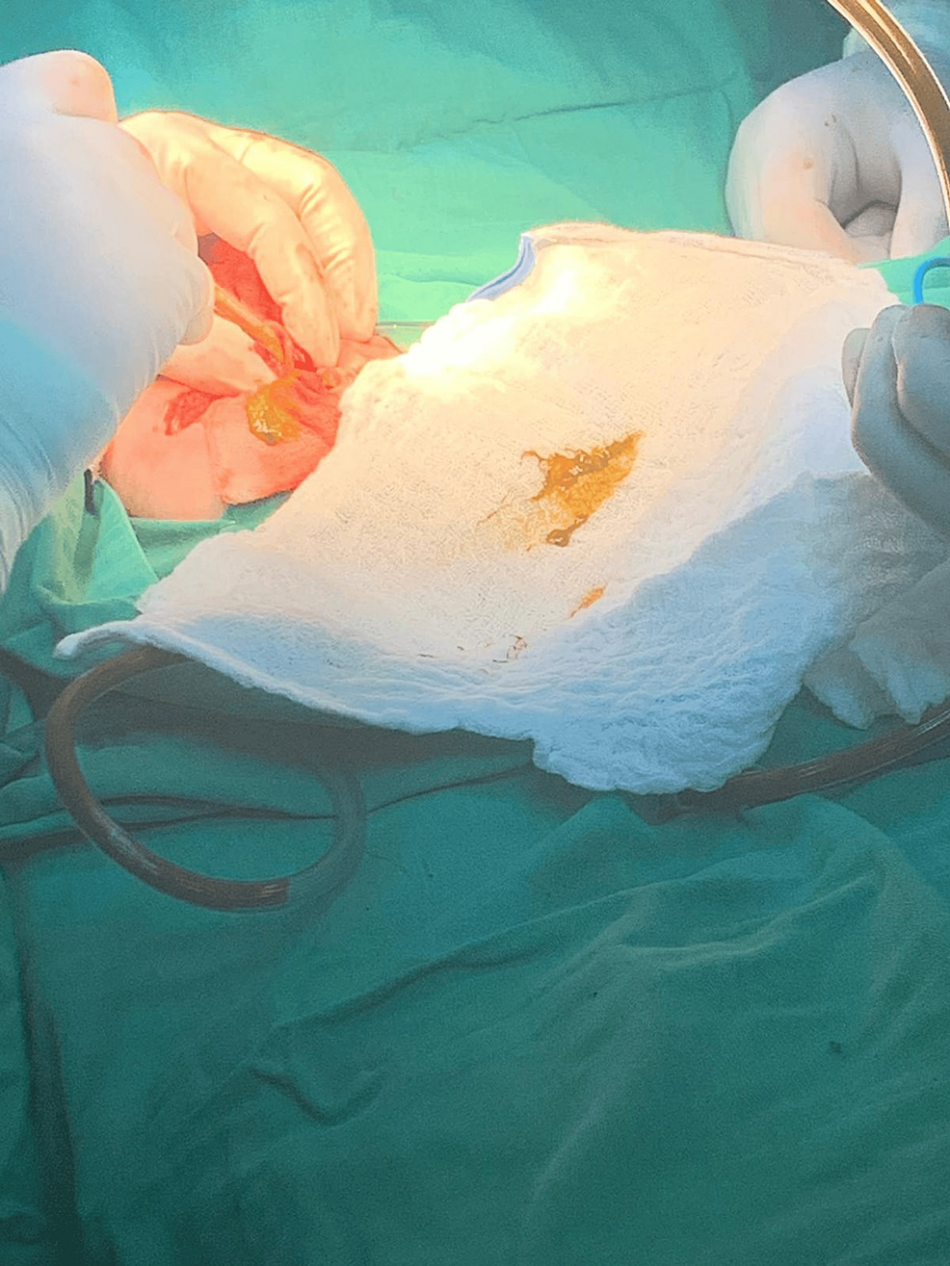
Intraoperative photograph showing suction of meconium that crammed above rectal stenosis

On the same day, the baby was sent for an exploratory laparotomy with the goal of surgically repairing the rectal blockage with ligation of the distal and proximal parts, which were opened by a sigmoid colostomy with excision of the atretic segment of the rectum. As shown in Figures 2-4, closure of colostomy with coloanal anastomosis by another laparotomy procedure usually occurs after six to eight weeks of colostomy surgery as long as baby weight becomes near 5 kg. Postoperatively, the patient was shifted to the NICU for observation. Ampicillin and Gentamicin were given, and fluids and IV analgesics were administered. The patient was vitally stable; the abdomen exhibited soft laxity, accompanied by a large horizontal scar on the right side with a colostomy. On the second postoperative day, stoma was functional and the patient developed short bowel syndrome, so the total parenteral nutrition was started to allow the patient to maintain adequate nutrition, and our pediatric GI doctor started her on oral feeding with neonate formula, metronidazole, zinc, and octreotide, initially resumed via orogastric tube (OGT), transitioning thereafter to sucking.

On the third postoperative day, the patient developed tachycardia, fever, and vomiting. Both the wound swab and blood culture were done and indicated the presence of *Klebsiella*. However, the urine culture showed no growth, and a lumbar puncture was not performed. Laboratory findings are shown in Table 3. Consequently, the patient was initiated on a 14-day course of Meropenem and received platelet transfusions accordingly.

**Table 3:** Third-day postoperation laboratory tests CRP: C-reactive protein.

Lab Test	Normal Range	Result
CRP	Less than 10 mg/L	137 mg/L
Platelet count	150,000-450,000 per microliter	42,000 per microliter

At the age of 67 days of the patient's life, she was discharged from the hospital against medical advice until the date of the replacement of the colostomy with end-to-end anastomosis. She was well, active, afebrile, off O_2_, off in vitro fertilization (IVF), feeding by sucking, had a colostomy, weight of 2,310 g, and all of her medical tests were within the normal range.

## Discussion

Jejunal atresia, a rare congenital anomaly occurring in approximately one in 12,000 live births, is characterized by the absence or narrowing of the jejunal lumen [10]. While maternal smoking and the use of vasoconstrictive drugs during pregnancy are recognized risk factors, the etiology remains multifactorial, often attributed to vascular accidents during intrauterine development [10]. These accidents may occur due to disruptions in blood supply to the intestines of the developing fetus, leading to malformation or narrowing of the jejunal lumen.

Clinically, jejunal atresia presents with symptoms such as bilious vomiting, abdominal distension, and delayed or absent passage of meconium. These symptoms reflect the obstructive nature of the condition and its impact on gastrointestinal function [10,11]. In our patient, the early onset of bilious vomiting and the inability to pass meconium highlighted the presence of a proximal obstruction, which prompted further investigation and diagnosis.

Prenatal ultrasound findings often reveal a dilated intestine with polyhydramnios, providing valuable clues for antenatal diagnosis [11,12]. Postnatally, radiographic imaging typically shows a characteristic double or triple bubble sign, indicative of upper intestinal obstruction [12]. This sign, along with clinical symptoms, aids in the diagnosis of jejunal atresia and guides subsequent management.

Surgical intervention, such as end-to-end anastomosis, is the primary treatment modality for jejunal atresia, aiming to restore intestinal continuity and function [10]. However, despite successful surgery, complications such as malabsorption, short bowel syndrome, strictures, and gastroesophageal reflux may occur, requiring ongoing monitoring and supportive care [10]. These complications can significantly impact the long-term health and quality of life of affected individuals, underscoring the importance of comprehensive postoperative management.

In our case, persistent symptoms following initial surgery led to further investigation and the diagnosis of rectal atresia, a rare high anorectal malformation characterized by obstruction proximal to the dentate line [6,7,12]. The co-occurrence of rectal stenosis with jejunal atresia in our patient represents an exceptionally rare clinical scenario, adding to the limited literature on rectal stenosis worldwide. This unique combination of anomalies poses significant diagnostic and management challenges, requiring multidisciplinary expertise and tailored treatment strategies.

Surgical management of rectal atresia typically involves colostomy with excision of the atretic segment followed by delayed anastomosis, aiming for optimal functional outcomes [6,7]. Close postoperative monitoring is essential to detect and address potential complications, ensuring the best possible long-term prognosis for the patient.

In conclusion, our case highlights the rare occurrence of congenital rectal stenosis and its unique association with jejunal atresia. The limitation in our case arises from the limited data in the literature, as the prognosis is yet to be known. This complex clinical scenario underscores the importance of comprehensive diagnostic evaluation and multidisciplinary management in neonates with complex gastrointestinal malformations. Further research and reporting of similar cases are warranted to enhance our understanding and management of these rare congenital anomalies.

## Conclusions

The premature neonate had intestinal obstruction and underwent laparotomy for jejunal atresia repair. Postoperatively, she developed vomiting and jaundice, revealing rectal atresia in a fluoroscopic study. The second laparotomy involved ligation and sigmoid colostomy creation. At the age of 67 days of the patient's life, she was discharged from the hospital against medical advice until the date of the colostomy closure with coloanal anastomosis procedure, which occurs after six to eight weeks from the colostomy surgery date, as long as the baby's weight is near 5 kg. In neonates with jejunal atresia, evaluating the lower gastrointestinal tract patency for rectal atresia is crucial to prevent misdiagnosis and minimize the number of surgeries required. It helps avoid attributing symptoms solely to intestinal obstruction and allows for a two-stage surgery approach, addressing both conditions in a single operation. Neglecting rectal stenosis while treating jejunal atresia can lead to delayed abdominal symptoms and an increased risk of necrosis and perforation.
